# A phase-variable capsule facilitates *Akkermansia muciniphila* colonization of the intestinal mucus layer

**DOI:** 10.1128/mbio.01048-26

**Published:** 2026-06-18

**Authors:** Liam Gracia, Elizabeth R. Hughes, Dustin R. Middleton, Katherine D. Mueller, Jessica A. Portillo, Agastya Sharma, Lauren Davey, Maria E. Panzetta, Rachael B. Chanin, Ami S. Bhatt, Raphael H. Valdivia

**Affiliations:** 1 Department of Integrative Immunobiology, Duke University 3065, Durham, North Carolina, USA; 2 Department of Molecular Genetics and Microbiology, Duke University 12277, Durham, North Carolina, USA; 3 Department of Medicine, Division of Hematology, Stanford University 6429, Stanford, California, USA; 4 Department of Genetics, Stanford University 6429, Stanford, California, USA; Universite de Geneve, Geneva, Switzerland

**Keywords:** capsule, phase variation, gut microbiome, mucin, biofilms

## Abstract

*Akkermansia muciniphila* is a mucophilic commensal bacterium that significantly impacts metabolic and immune homeostasis. However, the bacterial factors involved in colonization of the gastrointestinal tract are not well understood. Here, we clarify the role of capsular polysaccharides (CPS) in *Akkermansia* (*Akk*) colonization. We show that *Akkermansia* species have multiple *cps* loci, with *cps1* being the most conserved across species. We find that *cps1* is regulated by phase variation via invertible genetic elements, leading to population diversity during growth in culture media, mice, and humans. An *A. muciniphila* mutant that cannot produce capsular polysaccharides successfully colonized the intestines of mice but showed a reduced ability to interact with the mucus layer. Additionally, this mutant tended to form biofilms and increased aggregation on abiotic surfaces and within the gastrointestinal lumen. Our findings suggest that the *A. muciniphila* capsule acts as a phase-variable regulator of colonization by balancing planktonic mucus-associated states and biofilm formation.

**IMPORTANCE:**

*Akkermansia muciniphila*, a member of the human gut microbiota, is associated with improved metabolic and immune health. However, the bacterial factors that allow this organism to thrive in the intestine and interact with the host are not fully understood. We identify capsular polysaccharides as key regulators of *A. muciniphila* association with mucin-rich layers in the gastrointestinal tract and its proximity to the intestinal lining. Furthermore, capsule synthesis in Akkermansia is controlled by epigenetic switches; hence, a small but significant fraction of bacteria lack a capsule. These capsule-free bacteria cluster and are prone to forming biofilms. Therefore, capsular phase variation enables *A. muciniphila* to switch between different colonization states, underscoring the role of *A. muciniphila* glycans in adapting to the gut environment.

## INTRODUCTION

*Akkermansia muciniphila* is a gut commensal gram-negative bacterium that has been linked to host metabolic and immunological outcomes (1, 2). Higher abundance correlates with improved metabolic (3–5) and immunological (6, 7) health, but elevated levels of *A. muciniphila* are also associated with adverse outcomes (8, 9). These contrasting associations underscore the importance of understanding how *A. muciniphila* colonizes the gastrointestinal (GI) tract and how bacterial factors influence its location, persistence, and, ultimately, its impact on host physiology.

*A. muciniphila* is a mucin-degrading specialist that can use mucin as its sole carbon and nitrogen source (1, 10, 11). Mucins are a family of highly glycosylated glycoproteins, with MUC2 forming the main structure of the human intestinal mucus layer (12). The mucus layer acts as a viscoelastic barrier between the epithelium and intestinal contents and is rich in antimicrobial peptides and antibodies, providing additional barriers to limit microbial proximity to the host epithelium. The mucus layer in the small intestine is thin and easily penetrable. In contrast, the large intestine’s mucus layer is organized into an inner, dense, stratified layer attached to the epithelium and an outer, looser, unattached layer. Although the inner mucus layer often lacks bacteria, the microbiota can influence its overall composition (13, 14). The mucus layer offers a unique ecological niche in the GI tract that mucophilic commensals such as *A. muciniphila* can use as a nutrient source. In fact, *A. muciniphila* is commonly found in human mucosal biopsies (15). In mice, *Akkermansia* (*Akk*) is also concentrated in the mucus layer, but factors like diet and microbiota composition can affect its spatial distribution (16–20).

Surface polysaccharides, including capsular polysaccharides (CPS), govern microbial adhesion, aggregation, immune recognition, and overall community organization (21–29). In *Bacteroides*, distinct capsule types influence epithelial proximity, nutrient access, and immune modulation, thus contributing to persistence in the gut (23–26, 28–33). In *Escherichia*, capsules (K antigen) and the exopolysaccharide colanic acid influence biofilm architecture, stress tolerance, and host interactions (34–38, 39). For *A. muciniphila*, the composition of surface exopolysaccharides, with the exception of membrane lipooligosaccharides (40), is mainly unknown. *Akkermansia* genomes harbor multiple genes predicted to encode exopolysaccharide biosynthesis enzymes (41–45). These genes are differentially expressed under varying *in vitro* environmental conditions (44, 46–48) and are required for *A. muciniphila* fitness in the GI tract (49). In addition, previous reports support the presence of capsular polysaccharides in *Akkermansia* (1, 46, 50).

Here, we characterize how *Akkermansia* capsular polysaccharides vary across species and their roles in GI tract colonization. We identify *cps1*, a capsule biosynthesis locus conserved across *Akkermansia* species that undergoes phase variation and whose expression is regulated by growth in mucin. We find that the capsule enhances bacterial association with the mucus layer. Capsule-deficient mutants form aggregates in the gut lumen and are prone to forming biofilms. These findings suggest that the capsule is an important factor mediating *A. muciniphila* colonization and survival within the host mucus ecosystem.

## RESULTS

### *cps1* is a conserved capsule polysaccharide biosynthesis locus across *Akkermansia* species

**Fig 1:**
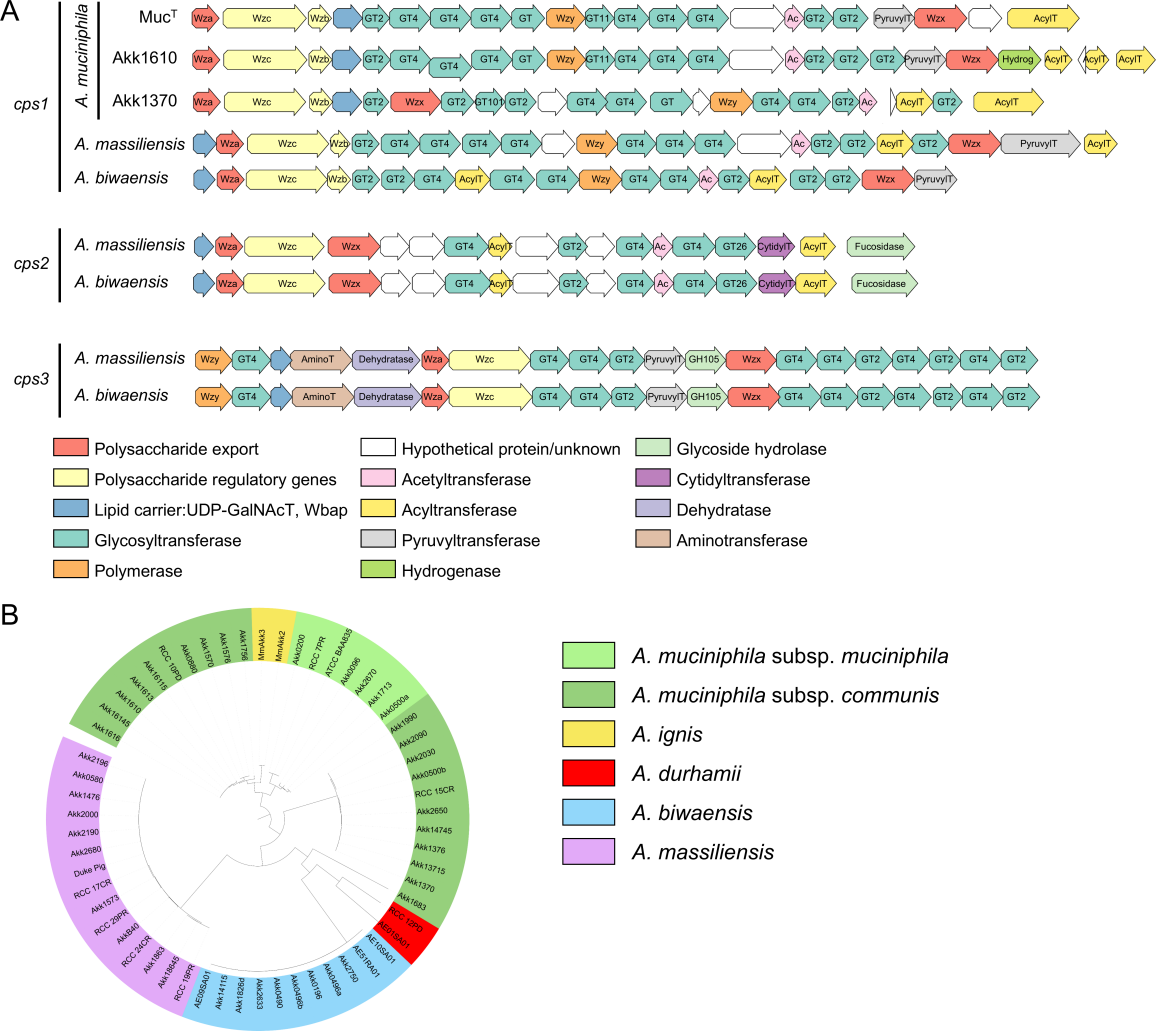
*Akkermansia* species harbor multiple capsular polysaccharide biosynthetic loci. (**A**) Genetic organization of predicted Wzx-Wzy capsular polysaccharide biosynthesis loci (*cps*) across *Akkermansia* strains. (**B**) Phylogenetic tree depicting relatedness of *cps1* across *Akkermansia* species.

*A. muciniphila* subsp. *muciniphila* Muc^T^ encodes a 26-kb locus (*amuc_2077–2098*), here designated *cps1*, with hallmark components of a Wzy-dependent capsule biosynthesis pathway, including a predicted export protein (Wza, Amuc_2077), polymerase (Wzy, Amuc_2086), and flippase (Wzx, Amuc_2096) (Fig. 1A; Table 1) (41–45). The presence of additional glycosyltransferases, acyltransferases, and chain-length regulators within this region is consistent with capsule assembly and export, as observed for group 1 capsule production in *Escherichia coli* (51).

**TABLE 1:** *Akkermansia* ATCC BAA-835/MucT *cps1* locus annotations

Gene	Function	CAZy family	Start	Stop
*amuc_2077*	Polysaccharide export outer membrane protein, Wza	na^*a*^	2,526,716	2,527,498
*amuc_2078*	Tyrosine-protein kinase, Wzc	na	2,527,629	2,530,031
*amuc_2079*	Capsular polysaccharide synthesis enzyme; manganese-dependent protein-tyrosine phosphatase, Wzb	na	2,530,094	2,530,765
*amuc_2080*	Lipid carrier: UDP-N-acetylgalactosaminyltransferase, Wbap	na	2,530,804	2,531,652
*amuc_2081*	Glycosyltransferase	GT2	2,531,678	2,532,454
*amuc_2082*	Glycosyltransferase	GT4	2,532,489	2,533,643
*amuc_2083*	Glycosyltransferase	GT4	2,533,640	2,534,818
*amuc_2084*	Glycosyltransferase	GT4	2,534,852	2,536,024
*amuc_2085*	Glycosyltransferase	GT	2,536,021	2,536,986
*amuc_2086*	Polymerase, Wzy	na	2,537,027	2,538,166
*amuc_2087*	Glycosyltransferase	GT11	2,538,156	2,538,986
*amuc_2088*	Glycosyltransferase	GT4	2,538,989	2,540,032
*amuc_2089*	Glycosyltransferase	GT4	2,540,032	2,541,171
*amuc_2090*	Glycosyltransferase	GT4	2,541,168	2,542,343
*amuc_2091*	Hypothetical protein	na	2,542,369	2,543,943
*amuc_2092*	Acetyltransferase	na	2,543,977	2,544,516
*amuc_2093*	Glycosyltransferase	GT2	2,544,520	2,545,368
*amuc_2094*	Glycosyltransferase	GT2	2,545,392	2,546,396
*amuc_2095*	Pyruvyltransferase	na	2,546,550	2,547,734
*amuc_2096*	Polysaccharide export protein, Wzx	na	2,547,727	2,549,262
*amuc_2097*	Hypothetical protein/unknown	na	2,549,302	2,550,306
*amuc_2098*	Acyltransferase	na	2,550,481	2,552,529

^*a*^ na, not applicable.

Because *Akkermansia* strains are genetically and phenotypically diverse (42, 52–55), we next examined whether *cps1* is conserved throughout the genus. We analyzed 58 genomes covering six species and subspecies (Table S1). All contained a predicted *cps1* locus (Fig. 1A), but phylogenetic analysis showed species- and subspecies-specific differences (Fig. 1B). Among *A. muciniphila* subsp. *communis* isolates, *cps1* clusters into two groups: strains like Akk1610, which carry nearly identical loci to the type strain Muc^T^ (subsp. *muciniphila*), and another group including strains like Akk1370, which display significantly different gene content and organization more similar to that in some *Akkermansia massiliensis* and *Akkermansia biwaensis cps1* loci, suggesting the potential to produce structurally different exopolysaccharides. Additionally, two more putative capsular polysaccharide biosynthesis loci (*cps2* and *cps3*) are present in *A. massiliensis* (Akk0580) and *A. biwaensis* (Akk0490). While the genes encoding WbaP, Wza, and Wzc are conserved across all *cps* loci, the additional genes in *cps2* and *cps3* loci show little sequence similarity to each other (Fig. S1), suggesting that *A. massiliensis* and *A. biwaensis* can produce two other distinct CPS structures, likely via a Wzy-dependent mechanism. Synteny analysis showed that *cps1* is located within a conserved chromosomal region across all species, between the cell division gene *ftsK* and a set of four genes predicted to encode a hypothetical protein, an aminoglycoside phosphotransferase, an L-fucose mutarotase, and a pyridoxal phosphate-dependent aminotransferase (Fig. S1). In several *A. muciniphila* strains (e.g., Akk1610, Akk1370, and MmAkk2), *cps1* is immediately next to prophages and tRNA loci, suggesting that this chromosome region is prone to horizontal gene transfer and may contribute to surface trait diversification.

### *cps1* is required for capsule formation in *A. muciniphila*

**Fig 2:**
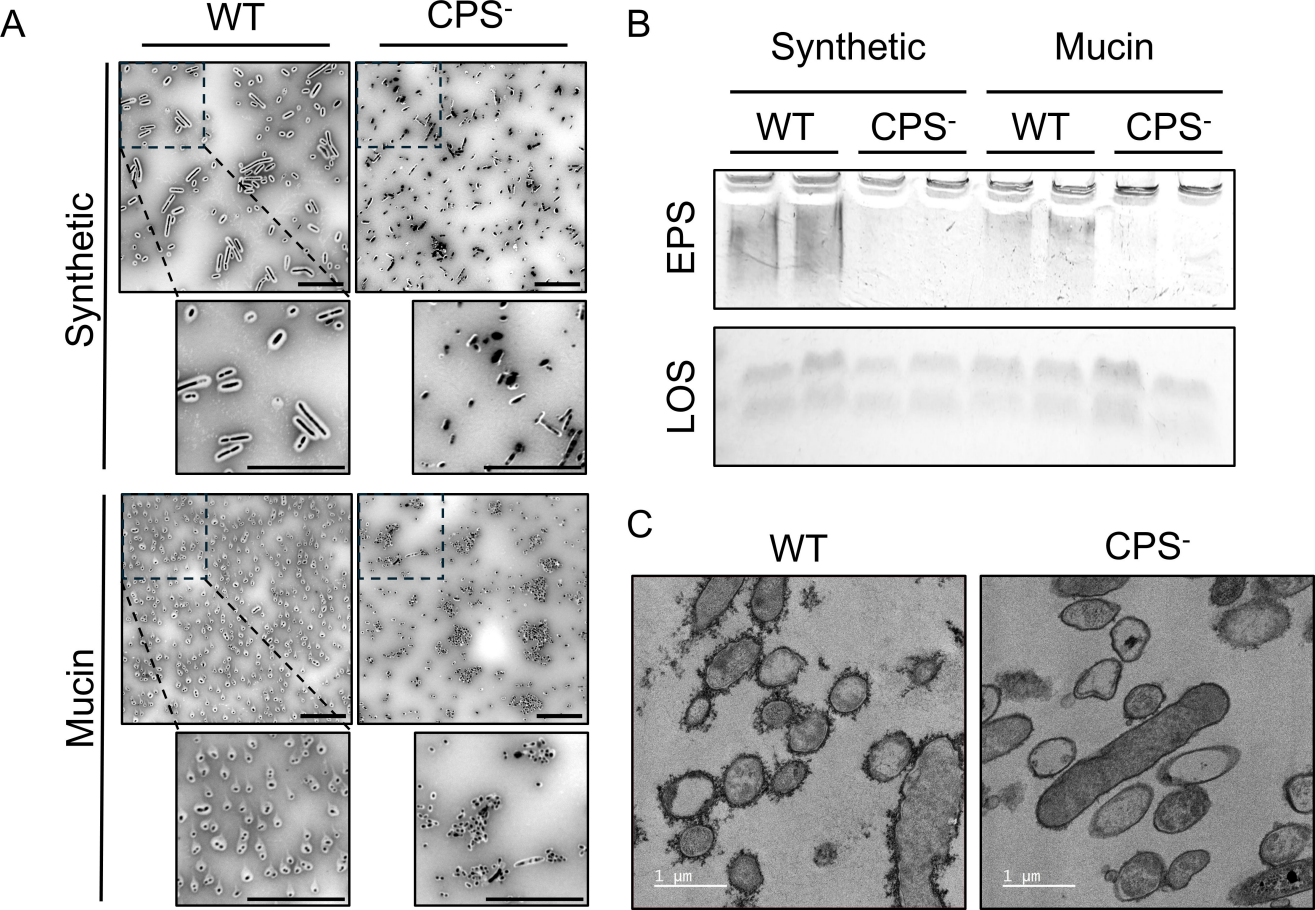
Capsule formation is Wzy-dependent in *A. muciniphila.* (**A**) Capsule production by wild-type (WT) *A. muciniphila* (strain Muc^T^) or an acapsular mutant (CPS^−^, *wzy*::Tn *gad*::Tn) was compared via Maneval’s staining. Capsules appear as zones of dye exclusion around bacteria. Scale bar, 20 µm. (**B**) Images of PAS-stained SDS-PAGE gels. EPS, exopolysaccharides. LOS, lipooligosaccharides. (**C**) Transmission EM images of ruthenium red-stained *A. muciniphila*. Scale bar, 1 µm.

To investigate capsule function, we isolated mutants with transposon (Tn) insertions in the *cps1* locus from a library of *A. muciniphila* Muc^T^ Tn mutants (49). A strain (CPS^−^) with Tn insertions in *amuc_2086*, encoding the predicted Wzy polymerase, and *amuc_0372* (*gad*) showed a loss of capsule as assessed by Maneval’s staining (Fig. 2A). Similarly, periodic acid-Schiff (PAS) staining of whole cell lysates resolved by SDS-PAGE indicated the absence of the high-molecular-weight polysaccharide band in this CPS^−^ strain compared to the wild-type strain (Fig. 2B). We stained *Akkermansia* cells with ruthenium red, a polycationic stain for negatively charged polysaccharides (56), and examined them by transmission electron microscopy (TEM) (Fig. 2C). Wild-type *A. muciniphila* Muc^T^, unlike the CPS^−^ strain, displayed a thick, electron-dense capsule surrounding the cell surface. We postulate that the residual electron-dense surface material seen on the CPS^−^ strain (Fig. 2C) is likely contributed by exopolysaccharides produced by a separate predicted exopolysaccharide biosynthesis locus (*amuc_1411-amuc_1414*) (41, 46, 48, 49, 57). The Tn insertion in the *gad* gene did not affect capsule production, as a separate strain with a single Tn insertion at this locus produced capsule (Fig. S2A and B). Furthermore, a mutant strain with a Tn insertion in *amuc_2077*, which encodes the predicted export protein Wza, also lacked a capsule (Fig. S2A and B). These results indicate that the *cps1* locus encodes the machinery required for capsule production, with *wzy* playing a critical role.

Based on TEM analysis, we found that an additional *A. muciniphila* subsp. *communis* strain Akk1370 also produces an electron-dense capsule after ruthenium red staining (Fig. S2C). In contrast, *A. massiliensis* and *A. biwaensis* showed little to no detectable electron-dense material. Unexpectedly, another *A. muciniphila* isolate (Akk1610 subsp. *communis*) showed no obvious capsule, indicating wide variation in capsule production among *Akkermansia* isolates.

### Capsule production is regulated during growth in mucin and is phase-variable

**Fig 3:**
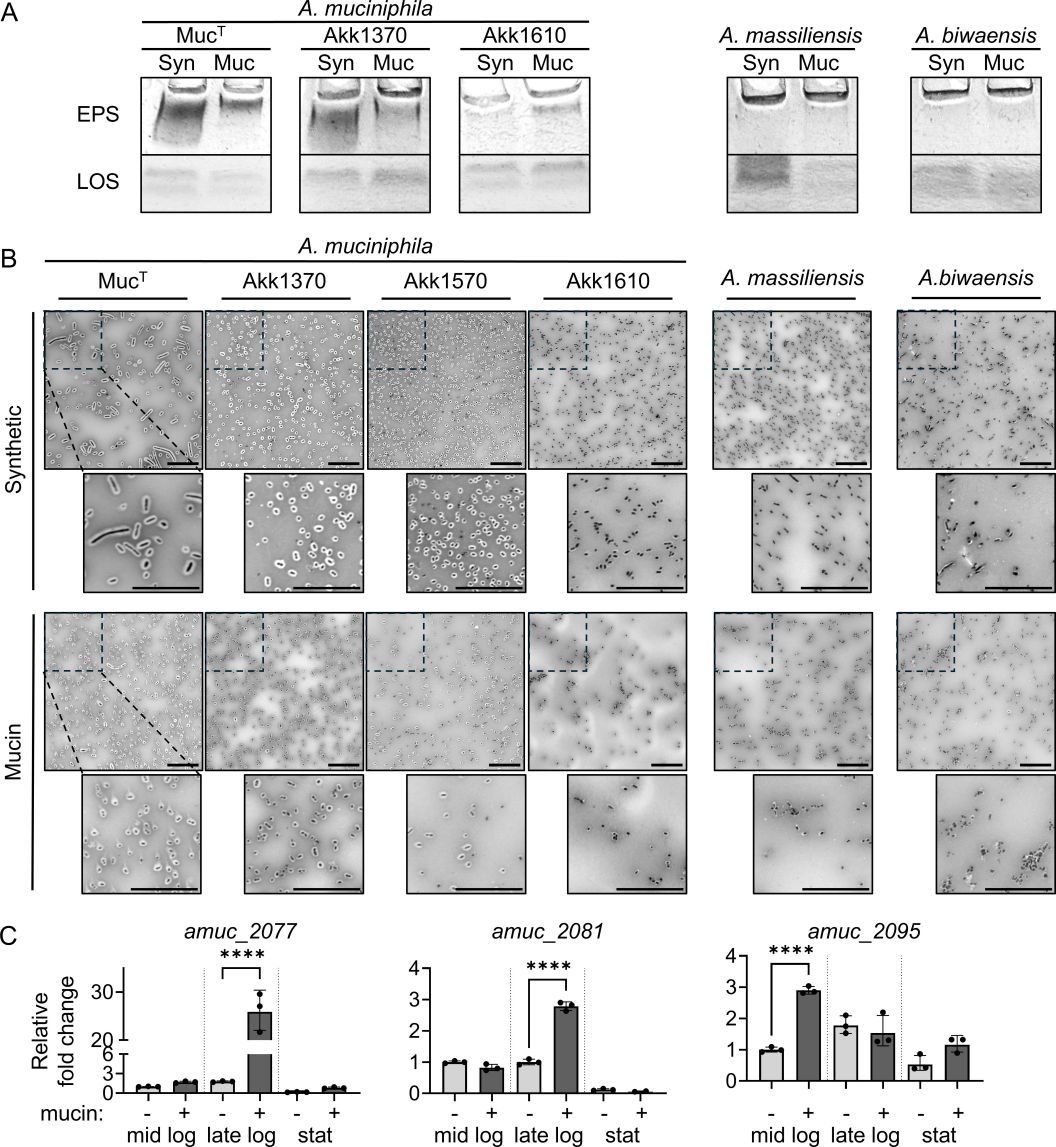
Growth in mucin media affects *A. muciniphila* CPS production. (**A**) Images of PAS-stained SDS-PAGE gels. EPS, exopolysaccharides. LOS, lipooligosaccharides. Syn, synthetic media (mucin-free). Muc, mucin media. (**B**) Images of Maneval’s staining of *Akkermansia* strains grown in synthetic or mucin media. Scale bar, 20 µm. (**C**) qPCR analysis of gene expression in *cps1* (*amuc_2077*, *amuc_2081*, and *amuc_2095*) across growth conditions from three biological replicates (symbols). Fold change is relative to the synthetic mid-log condition. Bars show geometric means ± geometric standard deviation. ****, *P* < 0.0001 based on an ordinary one-way ANOVA followed by Sidak’s multiple comparisons test. stat, stationary phase.

Because *Akkermansia* is a mucin-degrading specialist (1, 10, 11, 49), we tested whether growth in mucin media (with mucin as the only source of carbon and nitrogen) regulates capsule production. For both *A. muciniphila* strains Muc^T^ and Akk1370, exopolysaccharides from bacteria grown in synthetic or mucin media displayed different migration patterns in PAS-stained SDS-PAGE gels, suggesting changes in exopolysaccharide chain length or composition (Fig. 3A). In contrast, *A. massiliensis* and *A. biwaensis* displayed no detectable exopolysaccharides under either condition.

Maneval’s staining revealed thick zones of dye exclusion around *A. muciniphila* Muc^T^, Akk1370, and Akk1570 cells, indicating CPS production. In contrast, *A. muciniphila* strain Akk1610, *A. massiliensis,* and *A. biwaensis* produced little to no capsule (Fig. 3B). Growth media altered cell shape, with *A. muciniphila* Muc^T^ being more elongated in mucin-free synthetic media, consistent with previous findings that growth media impact *Akkermansia* cell size and morphology (1, 58, 59).

We then measured the transcription levels of three representative *cps1* genes from *A. muciniphila* Muc^T^: *amuc_2077* (*wza*), *amuc_2081* (a glycosyltransferase), and *amuc_2095* (a pyruvyl transferase). All three genes showed higher expression in cultures grown in mucin compared to synthetic medium during logarithmic growth (Fig. 3C; Fig. S3). Overall, these results suggest that mucin regulates capsule production and composition in *A. muciniphila*.

**Fig 4:**
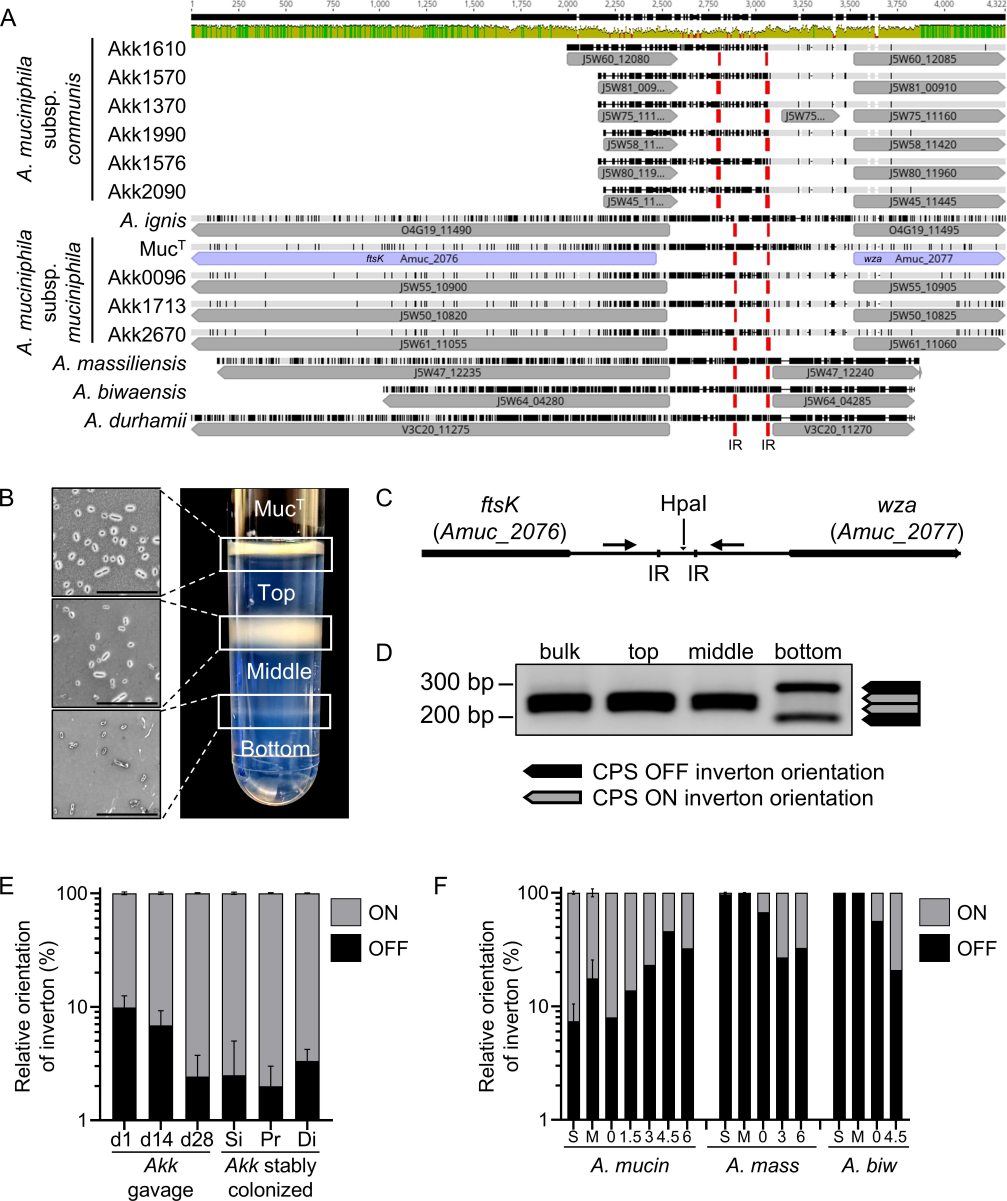
*A. muciniphila* CPS is phase-variable. (**A**) Alignment of region adjacent to *cps1* across *Akkermansia* strains. Red boxes correspond to IR. (**B**) Image of a Percoll gradient of synthetic-grown *A. muciniphila* Muc^T^ with images of Maneval’s staining from the layers. Scale bar, 20 µm. (**C**) Schematic of genomic region adjacent to *cps1* in *A. muciniphila* strain Muc^T^. Primers used to amplify inverton are depicted as arrows. IR, inverted repeat. (**D**) Assessment of inverton orientation across Percoll gradient layers by restriction digest of PCR products. (**E**) Assessment of the frequency of inverton orientation by qPCR in fecal samples from mice orally inoculated with *A. muciniphila* Muc^T^ (*Akk*) or in GI tract contents from mice stably colonized with *A. muciniphila* Muc^T^ via vertical transmission. Stacked bars correspond to mean ± SEM. d1, day 1 post-gavage; d14, day 14 post-gavage; d28, day 28 post-gavage; Si, small intestine (ileum); Pr, proximal colon; Di, distal colon. (**F**) Assessment of *cps1* inverton orientation in *A. muciniphila* strain Akk0096 (*A. mucin*), *A. massiliensis* (*A. mass*), and *A. biwaensis* (*A. biw*) from *in vitro* samples and fecal samples from humans naturally colonized with these *Akkermansia* strains. The predominant inverton orientation *in vitro* was assigned “ON” or “OFF” based on the observed presence or absence of CPS. Stacked bars correspond to mean ± SEM for *A. mucin* and *A. mass in vitro* samples (*N* = 3). Numbers (0, 1.5, 3, 4.5, and 6) correspond to the number of months post baseline collection at which human fecal samples were obtained. S, samples grown in synthetic media. M, samples grown in mucin media.

CPS production in gut commensals is often controlled by phase variation (60), as seen in *Bacteroides* spp., with invertible promoters regulating the expression of multiple CPS biosynthesis loci (28, 61). Verrucomicrobiota, including *Akkermansia*, contain a high number of invertible elements (invertons) (60, 62). We examined the region encompassing the *cps1* locus and identified an invertible element flanked by inverted repeats, consistent with the motif reported by Jiang and colleagues (60). Analysis of long-read *A. muciniphila* genomic sequences with PhaVa (62) revealed DNA sequencing reads that aligned to both inverton orientations. Based on an alignment of these chromosomal regions across *Akkermansia* strains, we determined that these inversion sites are conserved across the genus (Fig. 4A). Of note, Akk1610, which does not appear to form a capsule (Fig. S2C), has a prophage insertion adjacent to the *cps1* locus (Fig. S1) that truncates one of the inverted repeats in the inverton sequence.

To determine whether the capsule heterogeneity predicted by the *cps1* inverton is associated with phenotypic differences, we examined the cultures of *A. muciniphila* Muc^T^ for buoyancy variations on discontinuous Percoll density gradients. Density gradient ultracentrifugation has been used to explore natural variability in capsule production in *Bacteroides fragilis* and *Acinetobacter baumannii*, as well as capsule synthesis regulation in *Klebsiella pneumoniae* (63–69). Encapsulated bacteria tend to migrate poorly through dense Percoll solutions. We observed distinct banding patterns, indicating subpopulations with different buoyancies (Fig. 4B). Maneval’s staining of bacteria from each layer confirmed that cells in the upper fractions had capsules, while those in the lower fractions exhibited reduced dye exclusion. Next, we amplified the inverton-containing DNA region by PCR, followed by asymmetrical restriction enzyme digestion. This analysis showed that capsule-positive fractions were enriched for the “ON” orientation of the invertible element, whereas the capsule-negative fraction predominantly contained the “OFF” orientation (Fig. 4C and D).

To determine whether this variability also occurs during colonization of mammalian hosts, we examined fecal DNA from mice orally inoculated with *A. muciniphila* (Muc^T^) (Fig. 4E). The *cps1* inverton was predominantly in the “ON” orientation, although a small and consistent fraction of cells remained in the “OFF” state. The ratio of “ON” to “OFF” states was unchanged along the GI tract, with similar ratios of *cps1* inverton orientations observed in the small and large intestines of mice stably colonized with *A. muciniphila* Muc^T^.

We next tested stool samples from humans naturally colonized with *A. muciniphila* (strain Akk0096), *A. massiliensis*, and *A. biwaensis* and found that *cps1* invertons were also present in both orientations (Fig. 4F). Notably, for *A. massiliensis* and *A. biwaensis*, the predominant inverton orientation differed between bacteria grown in culture media and those found in feces, suggesting increased capsule production in the GI tract. Collectively, these findings demonstrate that phase variation in *Akkermansia* capsule expression occurs naturally in the mammalian gut and leads to the formation of distinct subpopulations with different surface properties.

### Capsule promotes association with the intestinal mucus layer

Insertion sequencing (INSeq) analysis of a pool of *A. muciniphila* Tn mutants (49) indicated that *cps1* provided a fitness advantage in the cecum of conventionally raised mice, but not in germ-free mice or during growth in mucin media (Fig. S4A). To assess the effect of *the A. muciniphila* capsule on colonization of the GI tract, we orally inoculated *Akkermansia*-free mice with either wild-type Muc^T^ or the CPS⁻ mutant (Fig. S4B). Both *A. muciniphila* strains successfully colonized the mice, with no statistically significant differences in fecal abundance between them 21 days after gavage (Fig. S4C). These results suggest that the capsule is not essential for survival in the GI tract but can provide a context-dependent competitive advantage.

**Fig 5:**
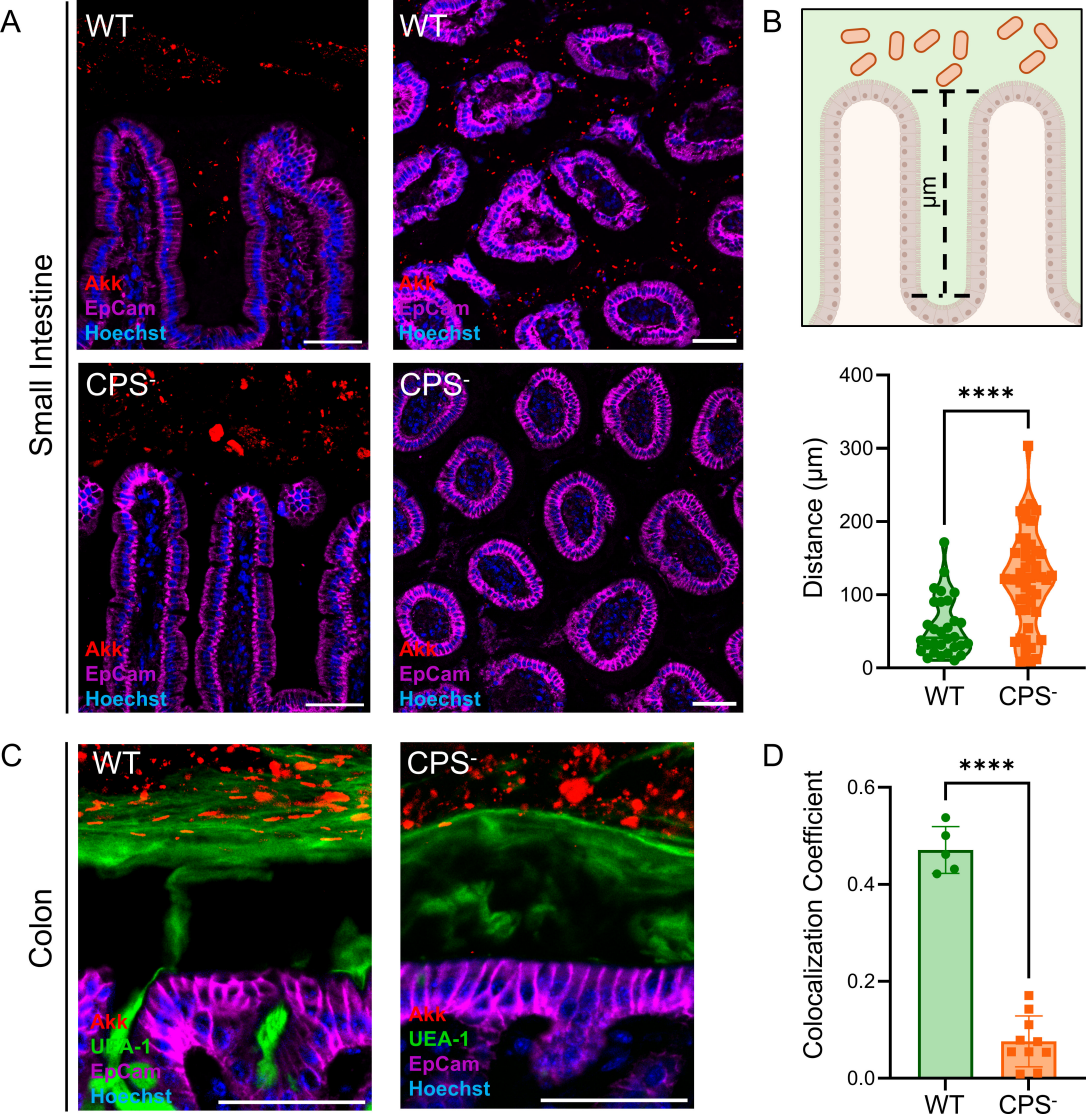
*A. muciniphila* CPS production facilitates colonization of the mucus layer. (**A**) Representative fluorescence images of *Akkermansia* in luminal sections from the small intestine of mice orally inoculated with wild-type (WT) or acapsular (CPS^−^) *A. muciniphila* (Akk). Left, transverse cross-sections with visible villi. Right, longitudinal cross-sections. Scale bar, 50 µm. (**B**) Schematic and quantification of distance of *Akkermansia* to the base of villi in the small intestine. Points represent individual measurements among tested mice. ****, *P* < 0.0001. (**C**) Transverse cross-sections of the mouse colon showing *Akkermansia* localized within UEA-1-labeled mucin. Scale bar, 50 µm. (**D**) Manders’ quantification of *Akkermansia* within UEA-1 mucin signal. Symbols correspond to data from individual mice. Bars represent mean ± SD. ****, *P* < 0.0001. Panel **B** was created with BioRender.

We observed clear differences in the spatial distribution within the mouse intestine between wild-type and CPS^−^ strains using indirect immunofluorescence microscopy. In the small intestine, wild-type bacteria were often found within the loose mucus layer near the epithelium, including between villi (Fig. 5A and B). In contrast, CPS^−^ *A. muciniphila* were rarely seen at these locations despite being plentiful in the intestinal lumen. In the colon, wild-type *A. muciniphila* was more frequently observed in association with the inner mucus layer (defined as condensed UEA-1-stained mucus) (14, 70), whereas CPS^−^ *A. muciniphila* mostly remained in the outer mucus layer and lumen (Fig. 5C and D). The decreased proximity to the epithelium and association with colonic mucus by the CPS^−^ strain suggests that the capsule is a key factor in mucus penetration.

The increased association of wild-type *A. muciniphila* with the inner mucus layer may be due to better penetration or increased survival at these sites compared to CPS^−^ mutants. Since antimicrobial peptides are concentrated within the inner mucus layer (71, 72), we examined whether the capsule protects *A. muciniphila* from LL-37, a human cationic antimicrobial peptide expressed in the ileum and colon (73). LL-37 treatment extended the lag phase in a dose-dependent manner, slowed the growth rate of wild-type *A. muciniphila*, and slightly decreased the maximum densities. In contrast, the growth of CPS^−^ bacteria was severely impaired (Fig. S5). This heightened sensitivity suggests that the capsule provides protection against host antimicrobial peptides, which may help explain the ability of wild-type *A. muciniphila* to survive within the inner mucus layer.

### Capsule-deficient *Akkermansia* forms aggregates and biofilms

**Fig 6:**
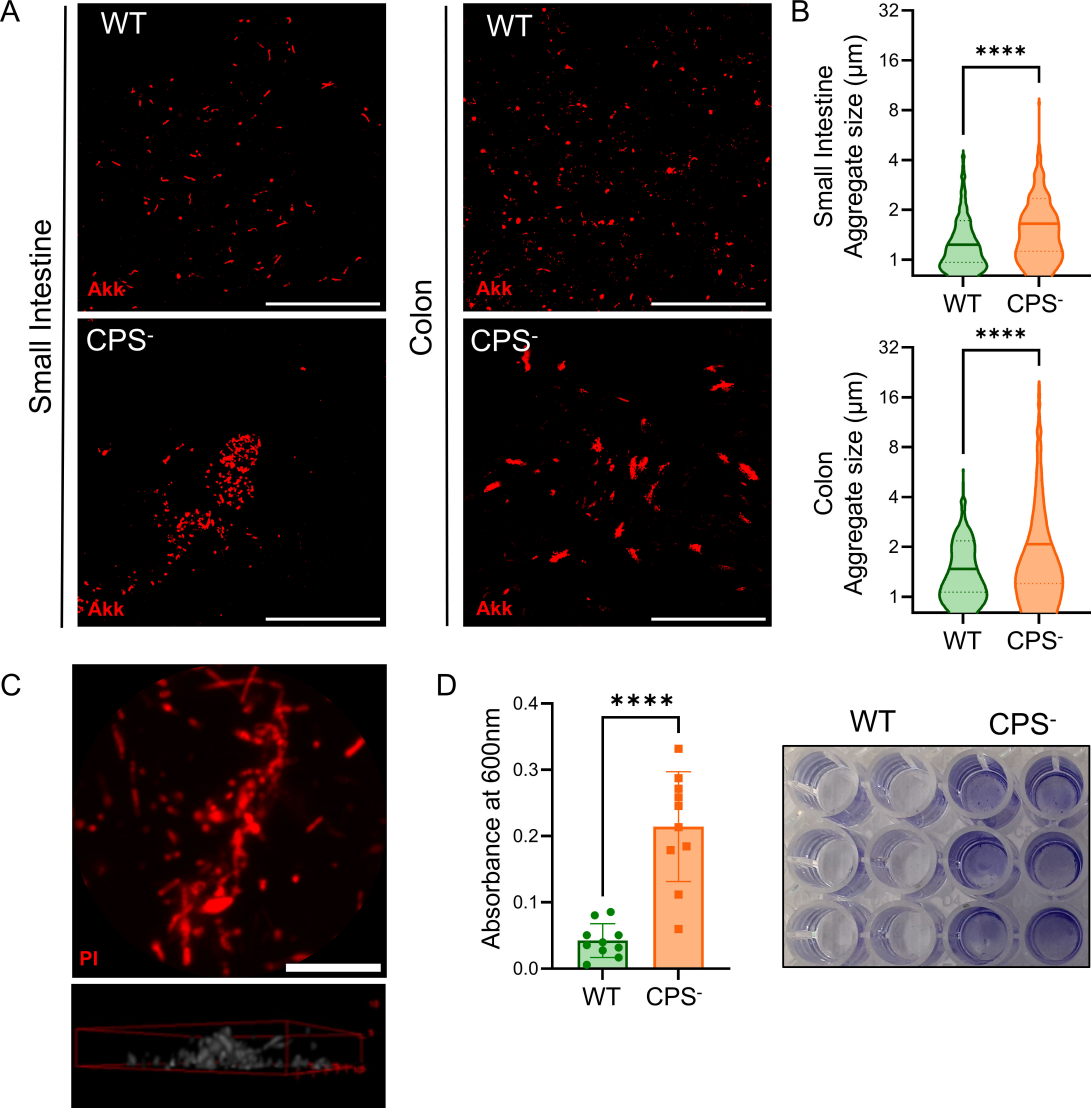
*A. muciniphila* CPS production reduces aggregation and biofilm formation. (**A**) Representative images of *Akkermansia*-stained lumen sections from the small intestine and colon of mice orally inoculated with WT or CPS^−^ *A. muciniphila* Muc^T^. Scale bar, 50 µm. (**B**) Quantification of *Akkermansia* aggregate sizes observed in lumen samples. Violin plots are truncated at 0.8 µm (approximate length of one bacterial cell). Medians correspond to solid lines and quartiles to dashed lines. (**C**) Image of propidium iodide (PI)-stained *in vitro* CPS^−^ biofilm. Scale bar, 10 µm. (**D**) Crystal violet assay of biofilm formation with representative image of plate. Symbols represent individual wells. ****, *P* < 0.0001. Akk, *Akkermansia*. WT, wild type. CPS^−^, acapsular mutant (*wzy*::Tn *gad*::Tn).

Loss of the capsule also changed *A. muciniphila’s* spatial distribution within the lumen of the small intestine and colon, with CPS^−^ mutants often forming multicellular aggregates (Fig. 6A and B). A measurement of aggregate sizes in the intestinal lumen showed a shift toward larger and more variable aggregates in the CPS^−^ strain compared to wild-type bacteria, which mostly remained dispersed.

Because the CPS^−^ mutant showed increased aggregation, we next investigated whether this phenotype reflected an inherent difference in surface association or matrix production. In biofilms, extracellular DNA is a key matrix component, and propidium iodide (PI) strongly stains it (74). When we compared PI staining of wild-type and CPS^−^ mutants grown anaerobically in synthetic media under static conditions, PI absorbance measured from the well bottom indicated that the aggregation seen in the intestinal lumen was replicated *in vitro*, and CPS^−^ bacteria formed three-dimensional biofilm structures (Fig. 6C). In contrast, wild-type cultures mostly remained planktonic and did not adhere to plates. Quantification using crystal violet staining further showed that the CPS^−^ mutant produced significantly more surface-adherent biomass than wild-type bacteria (Fig. 6D). These findings suggest that the absence of capsule promotes the transition to an aggregation-prone phenotype.

## DISCUSSION

Our results show that capsule production plays a key role in organizing *A. muciniphila* within the mucus layer (Fig. 5 and 6). By aiding colonization of the mucus layer (Fig. 5), the capsule supports the bacterium’s specialization as a mucin-degrading organism (10, 11, 49).

Phase-variable control of surface structures is a common strategy among gut bacteria to maintain diverse states that protect against sudden environmental changes, such as diet shifts, immune pressures, antimicrobials, or phage predation (28, 31, 60, 75–77). Consistent with this notion, we show that capsule expression in *Akkermansia* is phase-variable (Fig. 4). We described an invertible DNA region next to *cps1* that flips orientations to turn capsule production “OFF” or “ON.” Previous genetic analysis suggested that other mechanisms of phase variation in CPS1, such as slip-strand mispairing, may occur since long mononucleotide repeats are present at the end of *amuc_2098*, which encodes an acyltransferase in *cps1* (43).

**Fig 7:**
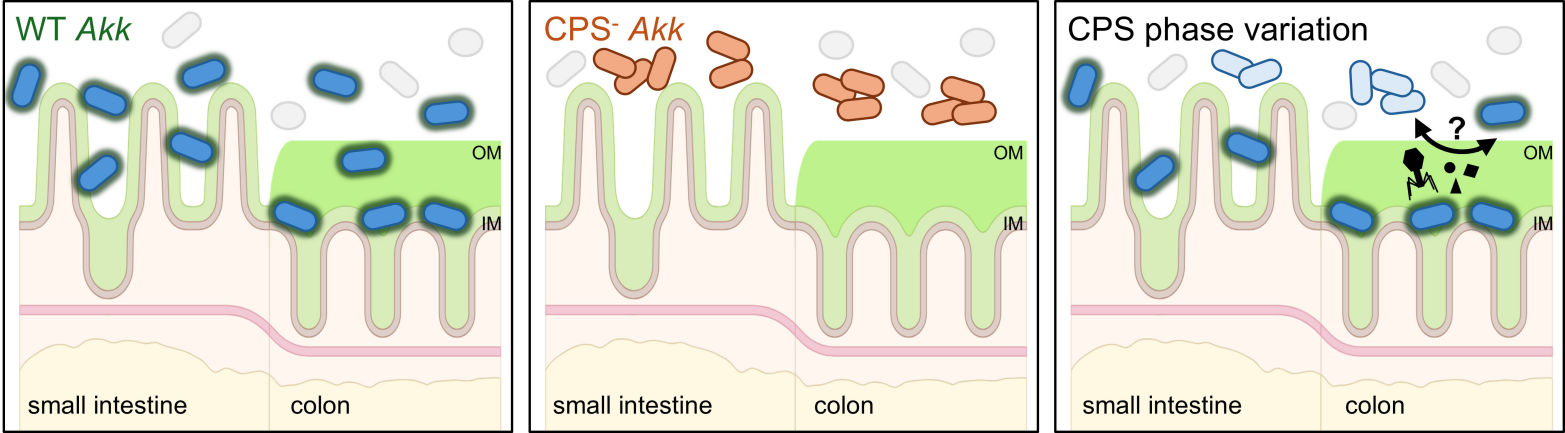
Model of the role of the capsule in *Akkermansia* colonization of the GI tract. Wild-type (WT) *Akk* colonizes the intervillous spaces in the small intestine and associates with the inner mucus layer in the large intestine, whereas acapsular (CPS^−^) mutants are restricted to the mucus periphery. These acapsular mutants form aggregates in the lumens of the ileum and colon. Because CPS expression is phase-variable, and both capsule and acapsular *Akkermansia* are naturally present in the gastrointestinal tract, we hypothesize that the relative abundance of these capsular states is driven by ecological pressures, including phage predation, exposure to antimicrobial peptides, and possibly biofilm-forming potential. OM, outer mucus layer; IM, inner mucus layer. Created with BioRender.

The tendency of acapsular bacteria to form biofilms and exhibit different spatial distributions in the GI tract has been observed in other gut bacteria (24, 29). *Bacteroides thetaiotaomicron* becomes more adherent and more likely to form biofilms in the absence of a capsule (29). For *B. fragilis*, a mutant lacking certain capsule types is defective in colonizing the proximal colonic mucus layer and produces fewer epithelial aggregates than the wild-type strain (24). We observed that an acapsular mutant of *A. muciniphila* was more adherent to abiotic surfaces and more prone to biofilm formation *in vitro* than wild-type bacteria (Fig. 6C and D), had difficulty colonizing the inner mucus layer (Fig. 5), and formed aggregates in the intestinal lumen (Fig. 6A and B). These findings support a model (Fig. 7) in which capsule “ON” *Akkermansia* cells are concentrated in mucus near the epithelium, thereby protecting them from higher levels of antimicrobial peptides (Fig. S5) and other ecological stresses. Conversely, capsule “OFF” cells may serve different ecological roles, such as resistance to phages. The frequent phage insertions near the *cps1* locus (Fig. S1) suggest that phages target the *Akkermansia* capsule. In *Bacteroides* spp., phage infections and host immune responses influence CPS phase variation and types (28, 31, 78, 79).

The expression of representative *cps1* genes increased during logarithmic growth in mucin compared to synthetic media (Fig. 3C). Expression of *cps1* genes in *A. muciniphila* has been proposed to be regulated by factors such as oxygen exposure (47), polyphenols (46), and different growth media (44, 48). Although previous studies suggested that *cps1* was downregulated during growth in mucin (44, 48), we found that the mucin-dependent increase in *cps1* expression highly depends on the bacterium’s growth phase (Fig. S3). Differences in experimental setup may also explain the variation in findings. We did not observe any notable differences in capsule thickness between the two growth conditions, but exopolysaccharides showed distinct migration patterns on SDS-PAGE (Fig. 3A and B). These results imply that changes in polymer processing, turnover, CPS chain length, or composition might occur between growth conditions, which aligns with other bacteria where exopolysaccharide production and chemistry change with carbon source and stress (80–87).

Bacterial residence within the mucus layer confers an ecological advantage to specific gut microbes, including those capable of degrading mucin glycans for their metabolic needs. However, this niche also poses significant challenges to colonization. Mucus contains high levels of secretory IgA (88), antimicrobial peptides (71–73), and bacteriophages (89). Our findings that the capsule promotes the association of *A. muciniphila* with mucin-rich regions support a model in which the capsule aids survival and persistence within the mucus layer by providing a protective barrier that shields cells from antimicrobial peptides and other ecological pressures in the mucus layer. Simultaneously, capsular polysaccharides can serve as targets for phages, which may explain why *Akkermansia* species encode multiple *cps* loci and regulate capsule expression through phase variation. *Akkermansia* cells that lack capsule production tend to form more aggregates and biofilms, both *in vivo* and *in vitro*, possibly representing an alternative strategy to improve survival outside mucus-associated niches and promote transmission.

## MATERIALS AND METHODS

### Bacterial strains and growth conditions

*Akkermansia* strains were grown in an anaerobic chamber (5% hydrogen, 5% carbon dioxide, 90% nitrogen; Coy Laboratory Products, Grass Lake, MI) at 37°C. Strains were grown in mucin, synthetic, synthetic + mucin, or BHI + mucin media, with agar added to make plates (1.25%). Details on the strains used and medium composition are provided in the Supplemental Materials and Methods.

### Prediction of capsular polysaccharide biosynthesis loci

We assigned predicted functions of genes within Muc^T^ (NCBI Genomic Sequence NC_010655.1) *cps1* in Table 1 using a combination of NCBI annotations, glycosyltransferase assignments with dbCAN3 (90), and comparisons of predicted protein structures to PDB using Phyre2 (91) and AlphaFold (92, 93). To identify additional *cps* loci in other *Akkermansia* strains, we performed Tblastn using the Amuc_2077 protein-coding sequence (WP_012421100.1). We examined the annotations and genetic organization of the surrounding genes and assigned predicted functions (Fig. 1A).

### Phylogenetic analysis of the *cps1* biosynthesis locus

Comparison of the *cps1* locus among 58 *Akkermansia* genomes (Table S1) was performed using the anvi’o version 7.1 pangenomic workflow (94). Details regarding the workflow are provided in Supplemental Materials and Methods.

### Synteny analysis of *cps*

The genetic sequences of *cps* loci and their flanking regions were retrieved from the NCBI and put into the CAGECAT web server (95) to generate gene cluster comparison figures. Annotations were manually added in Adobe Illustrator using the tRNA annotations from NCBI, assignments of *cps* loci based on NCBI gene annotations as described above, and predicted prophages based on similarities between the inserted DNA sequences and metagenome-assembled phage genomes (by BLASTN).

### Analyses of exopolysaccharides

*Akkermansia* exopolysaccharides were analyzed by Periodic–acid Schiff staining of cell pellets run on gels, transmission electron microscopy imaging of ruthenium red-stained cells, Maneval’s staining, and Percoll gradient density analysis. Method details are provided in the Supplemental Materials and Methods.

### Analysis of *A. muciniphila* Muc^T^ *cps* gene expression by RT-qPCR

Starter cultures were grown for 3 days in cysteine-supplemented synthetic medium from glycerol stocks. Cultures were diluted to starting OD_600_ = 0.05 into 24-mL cysteine-supplemented synthetic or mucin medium. OD_600_ was measured at the indicated times. Bacteria were harvested by centrifugation (10,000 × *g*, 5 min, 4°C) and resuspended in TRIzol (Invitrogen, Waltham, MA; #15596026). RNA extraction was performed according to the TRIzol User Guide. RNA was incubated with DNase I (NEB, Ipswich, MA) at 37°C for 10 min. DNase was inactivated by adding EDTA (5 mM final) at 75°C for 10 min. cDNA was synthesized using the iScript cDNA kit (Bio-Rad, Hercules, CA) with 2 µL of DNase-treated RNA in a 20-µL reaction per the manufacturer’s protocol. Relative expressions of *amuc_2077*, *amuc_2081*, and *amuc_2095* were measured by SYBR Green qPCR, normalizing to 16S rRNA using the comparative Ct method. Reactions contained 2 µL of 1:5 diluted cDNA and 375 nM primers in a 10-µL total volume. Primer sequences are in Table S3. qPCR was performed on a QuantStudio 6 Pro (Life Technologies, Carlsbad, CA). One mucin-stationary sample was excluded due to high technical replicate variation (SD > 0.5).

### Alignment of the region adjacent to *cps1*

Inverted repeats were manually annotated based on the motif identified by Jiang and colleagues (CGGATT..AATCCG) (60). Sequences adjacent to the first gene in the *cps1* locus were aligned in Geneious (version 2025.0 created by Biomatters; available from https://www.geneious.com/) using Clustal Omega 1.2.2 with fast clustering (mBed algorithm); a cluster size of 100 was used for mBed Guide Trees, and sequences were grouped by similarity.

### DNA extraction

DNA was extracted from *in vitro* cultures and Percoll gradient layers with the DNeasy Blood and Tissue Kit (Qiagen, Hilden, Germany), following the manufacturer’s recommendations for pretreatment for gram-negative bacteria. DNA was extracted from mouse samples with the DNeasy PowerSoil Pro Kit (Qiagen, Hilden, Germany), according to the manufacturer’s recommendations. DNA from human fecal samples was previously extracted with the QIAamp Fast DNA Stool Mini Kit (Qiagen, Hilden, Germany; #51604) (45). Human fecal samples were obtained from the POMMS Biorepository (Clinical trial identifier # NCT03139877) (96). The Duke Institutional Review Board approved the study design and procedures. Details are in the Supplemental Materials and Methods.

### Analysis of inverton orientation

The inverton upstream of *cps1* in MucT was identified using the long-read inverton predictor PhaVa (62). All publicly available *A. muciniphila* long-read sequencing data sets deposited on Sequence Read Archive (eight total as of May 2023) were run through the PhaVa pipeline using *A. muciniphila* ATCC BAA-835 as the reference genome. Putative invertons in other *Akkermansia* strains were identified by manually searching genomes adjacent to *cps* loci for inverted repeat sequences consistent with the motif identified by Jiang and colleagues (CGGATT..AATCCG) (60). Inverton orientations were analyzed by restriction enzyme digest and qPCR. Details are provided in the Supplemental Materials and Methods.

### INSeq data graphs

INSeq data (49) were plotted with ggplot2 (version 3.5.2) in R (version 4.5.0), and images were edited in Adobe Illustrator 2026.

### Colonization of mice with *Akkermansia*

Male and female 6- to 18-week-old C57BL/6J SPF mice were used. Animals were obtained from The Jackson Laboratory (Bar Harbor, ME) or bred at Duke University. Mice were maintained on a 12-h light/dark cycle and had access to food and water *ad libitum*, except in approved cases of fasting prior to gavage. Details regarding the animal experiments are provided in the Supplemental Materials and Methods.

### Quantification of *A. muciniphila* colonization by qPCR

*Akkermansia* absolute abundance was determined via the standard curve method using SYBR Green-based qPCR, as described previously (49) with *Akkermansia*-specific primers (Table S3) using PowerUp SYBR Green Master Mix (Thermo Fisher Scientific, Waltham, MA) on a QuantStudio 6 Pro real-time PCR system (Applied Biosystems, Waltham, MA). *Akkermansia* abundance was calculated as genome equivalents per gram of fecal material.

### Confocal microscopy

Tissues were fixed in Carnoy’s solution and stained with anti-*Akkermansia* antisera, anti-EpCAM, UEA-1 Fluorescein, and Hoechst. Images were acquired on a Zeiss LSM 88. Additional details regarding fixation, staining, and imaging are available in the Supplemental Materials and Methods.

### LL-37 growth curve assay

Overnight cultures of wild-type and CPS^−^ *A. muciniphila* Muc^T^ were cultured 1:20 into fresh synthetic medium supplemented with LL-37 (Cayman Chemical Company, Ann Arbor, MI; #24461) at 0 (control), 10, or 20 μM. Cultures were dispensed into 96-well plates (150 μL per well overlaid with 50 μL paraffin oil, *N* = 5 per strain and condition) and incubated anaerobically at 37°C for 96 h with OD_600_ measured every hour in a plate reader (SPECTROstar Nano; BMG Labtech, Cary, NC).

### Biofilm assays

*Akkermansia* cultures were grown in synthetic media to assess biofilm formation using a crystal violet assay or confocal imaging of propidium iodide-stained biofilms. Details are available in the Supplemental Materials and Methods.

### Statistical analysis

GraphPad Prism version 10.6.1 was used for statistical analysis and plot generation. For statistical analysis of gene expression changes (Fig. 3C), ordinary one-way ANOVA followed by Holm-Sidak’s multiple comparisons test, with a single pooled variance, of preselected columns was performed. All other statistical analyses used Welch’s *t*-test. *P* values of less than 0.05 were considered statistically significant.

## SUPPLEMENTAL MATERIAL

Supplemental Material mbio.01048-26-s0001.pdf Supplemental figures and expanded methods.

Supplemental Tables mbio.01048-26-s0002.xlsx Tables S1-S3.

ASM does not own the copyrights to Supplemental Material that may be linked to, or accessed through, an article. The authors have granted ASM a non-exclusive, world-wide license to publish the Supplemental Material files. Please contact the corresponding author directly for reuse.
