# Supplementary material for: A phase-variable capsule facilitates Akkermansia muciniphila colonization of the intestinal mucus layer
Source: mBio. 2026 Jun 18;17(7):e01048-26. doi: 10.1128/mbio.01048-26 (PMC13343947; doi:10.1128/mbio.01048-26)

## SUPPLEMENTAL FIGURE LEGENDS

**Figure S1. Synteny analysis of *cps1-3* loci.** *cps*, capsular polysaccharide loci. Scale bar, 2.5 kb. 1, cell division gene *ftsK*; 2, gene encoding a hypothetical protein; 3, aminoglycoside phosphotransferase; 4, L-fucose mutarotase; 5, pyridoxal phosphate-dependent aminotransferase

**Figure S2. Capsule formation in *A. muciniphila* is Wzy- and Wza-dependent.** Percoll gradients (A) and Maneval's staining (B) of *wza*::Tn (*amuc_2077*::Tn) and *gad*::Tn (*amuc_0372*::Tn) mutants of $Muc^T$. Scale bar, 20 μm. (D) TEM images of ruthenium red-stained *Akkermansia* strains. Arrows indicate electron-dense CPS on the bacterial surface. Scale bar, 200 nm.

**Figure S3. Growth curve for samples used in gene expression analysis.** Arrows indicate the time points at which samples were collected for gene expression analysis. Symbols represent each biological replicate. Curves correspond to logistic growth, least-squares fit.

**Figure S4. INSeq analysis and single colonization experiments of mice with *A. muciniphila*.** (A) Volcano plots from publicly available INSeq datasets (1). Gray symbols: mutants present in the dataset. Black symbols: mutants with transposon insertions in *cps1* genes. SPF: specific-pathogen-free. (B) Schematic of the experimental design showing oral gavage of mice with either wild-type (WT) or acapsular ($CPS^-$, *wzy*::Tn, *gad*::Tn) *A. muciniphila*. *Akk, Akkermansia*. (C) Quantification by qPCR of *Akkermansia* levels in fecal

samples collected 21 days after oral inoculation. Akk-, *Akkermansia*-free mice. Symbols represent data from individual mice. Bars represent geometric means ± geometric standard deviation. ns, not statistically significant.

**Figure S5. Growth kinetics of *A. muciniphila* in the presence of LL-37.** Growth curves of wild-type ($Muc^T$) and acapsular ($CPS^-$) *A. muciniphila* cultured in synthetic media containing the antimicrobial peptide LL-37. Each point represents a replicate, and bars show mean ± SD.

## SUPPLEMENTAL MATERIALS AND METHODS

**Bacterial strains and growth conditions.** BHI+mucin media is prepared from BD Bacto Brain Heart Infusion broth (BD, Franklin Lakes, NJ) supplemented with 0.25% porcine gastric mucin (PGM) [type III, Sigma-Aldrich, Darmstadt, Germany; autoclaved]. Mucin media, based on previous work (2), consists of 3 mM $KH_2PO_4$, 3 mM $Na_2PO_4$, 5.6 mM $NH_4Cl$, 1 mM $MgCl_2$, 1 mM $Na_2S \cdot 9H_2O$, 47 mM $NaHCO_3$, 1 mM $CaCl_2$, 40 mM HCl, trace elements and vitamins, and 0.25% PGM. For synthetic media, PGM was replaced with 0.2% GlcNAc (filter-sterilized), 0.2% glucose (filter-sterilized), 16 g/liter soy peptone, and 4 g/liter threonine (autoclaved) (1). For synthetic+mucin media, PGM (0.25%) is added to synthetic media. Media was supplemented with filter-sterilized L-cysteine to a final concentration of 0.5 mM, as indicated. When required, antibiotics were used at the following concentrations: 7 ug/ml chloramphenicol, 10 ug/ml gentamicin, and 12 ug/ml kanamycin.

*Akkermansia* strains used in this study are listed in Table S2. *A. muciniphila* $Muc^T$ was obtained from ATCC (BAA-835). Additional human *Akkermansia* strains were isolated previously (3) as part of the POMMS study (4). Unless indicated otherwise, MmAkk2, Akk0580, Akk0490, and RCC_12PD were used as representative strains of *A. ignis*, *A. massiliensis*, *A. biwaensis*, and *A. durhamii*, respectively. Transposon insertion mutants in strain $Muc^T$ were previously described (1) and isolated from the arrayed library on agar plates containing chloramphenicol. The transposon insertion in *amuc_2086* was confirmed by PCR using the primers listed in Table S3. Bacterial genome sequencing was performed by Plasmidsaurus (Louisville, KY) using Oxford Nanopore Technology, with custom analysis and annotation. The $CPS^-$ strain had transposon insertions in *amuc_2086* (*wzy*) and *amuc_0372* (*gad*), whereas the *amuc_0372*::Tn (*gad*::Tn) and *amuc_2077*::Tn (*wza*::Tn) strains had only one transposon insertion site in the indicated genes.

**Study approval for human samples.** Recruitment of participants and handling of clinical samples were approved as part of the Pediatric Obesity Microbiome and Metabolism Study (POMMS; Clinical trial identifier #NCT03139877), as described in detail in (4) and approved by the Duke Institutional Review Board in document Pro00074729. Written informed consent was obtained prior to participation and before the inclusion of serum and fecal samples for further study in the POMMS biobank, which was governed by a separate IRB protocol, Pro00074546. Data, serum, and stool samples from the study participants have been banked and are available as a repository (Clinical trial identifier #NCT02959034) for further research, as described at https://sites.duke.edu/pomms. The

POMMS Biorepository was the source of the *Akkermansia* isolates described above (Table S2, (3)) and of stool samples from humans naturally colonized with *Akkermansia* used to investigate *cps1* inverton orientation (Fig. 4F).

**Phylogenetic analysis of the *cps1* locus.** To satisfy anvi'o formatting requirements, the fasta files containing the indicated genomes (Table S1) were converted into contigs-fasta format using anvi-script-reformat-fasta with parameters --simplify-names and --seq-type NT. Genome databases were then generated using the reformatted genomes using anvi-gen-contigs-database. The resulting databases were annotated using anvi-run-hmms and anvi-run-cogs (5) and used to create a genome storage database using anvi-gen-genomes-storage. A pangenome was constructed using anvi-pan-genome with parameters --minbit 0.5, --mcl-inflation 10, and --use-ncbi-blast. For comparison of sequences across *Akkermansia* genomes, *cps1* was defined as gene caller IDs corresponding to *amuc_2077* through *amuc_2100*. Gene caller IDs were defined by anvi'o, and inspection of the HMM annotations revealed that *amuc_2077* was identified as Wza, and *amuc_2100* was identified as either SrkA or CotS, depending on the genome.

Sequences were extracted through a three-step process, each using anvi-export-locus. In the first step, default mode was used to extract 100 gene caller IDs upstream and 40 gene caller IDs downstream of Wza. The second step used the flank mode with the search terms Wza and CotS. The third step used the flank mode with the search terms Wza and SrkA. The resulting fasta files from the second and third steps were combined to represent

the *cps1* locus across all *Akkermansia* genomes. The sequences were aligned, and a dendrogram was generated using Clustal Omega (6). The dendrogram was visualized with Interactive Tree of Life and edited in Inkscape (7).

**Maneval's staining of *Akkermansia*.** Bacteria from liquid cultures or Percoll gradient layers were washed in PBS before staining. Five μL of bacterial suspension was mixed with 5 μL of 1% w/v Congo red (Sigma-Aldrich, St. Louis, MO; #C6767) and spread onto slides. After air drying, slides were flooded with Maneval's solution (Carolina Biological Supply Company, Burlington, NC) for 5 minutes, rinsed with water, and air dried. Slides were imaged on the Axio Imager Z2 upright microscope (Carl Zeiss Microscopy GmbH, Germany).

**Periodic Acid-Schiff staining of gels to detect exopolysaccharides.** *Akkermansia* was grown in mucin or synthetic medium (cysteine-supplemented for Fig. 3A). Cell quantities were normalized by OD600, pelleted, and boiled in loading dye for 5 minutes. Samples were vortexed, centrifuged at 20,000 g for 10 minutes, and run on 4–20% or 4–15% gradient gels (Bio-Rad, Hercules, CA; #4568123 or #4568083). Gels were washed in water, fixed in 12.5% trichloroacetic acid for 20–30 minutes, oxidized in 1% periodic acid and 3% acetic acid for 1 hour (protected from light), washed in water for 4 hours to overnight, stained with Schiff's reagent for 1 hour, and reduced with 0.5% sodium metabisulfite three times for 10 minutes each. Gels were washed and imaged.

**Transmission electron microscopy (TEM) imaging of ruthenium red-stained *Akkermansia*.** *Akkermansia* strains were cultured on BHI agar plates supplemented with 0.25% PGM. Bacterial lawns were scraped into 400 µl of PBS and pelleted (3 min, 15,000 × g). Pellets were fixed for 1 h on ice in 2% glutaraldehyde, 2% paraformaldehyde, 0.075 M lysine-acetate, and 0.075% ruthenium red in 0.1 M cacodylate buffer, then washed twice for 15 min in buffer containing 0.15% ruthenium red. Samples were post-fixed in 1% osmium tetroxide with 0.15% ruthenium red for 1 h at room temperature and washed twice in the same buffer. Pellets were dehydrated through graded acetone (30–100%), followed by two 100% ethanol rinses (15 min each), then incubated at 4°C overnight. Samples were infiltrated with Spurr's resin via 25%, 50%, 75%, and 100% resin/acetone steps and polymerized at 70°C for 72 h. Ultrathin (60 nm) sections were cut with a Diatome diamond knife, collected on slot grids, stained with uranyl acetate and lead citrate, and imaged on a FEI Tecnai G2 Twin TEM at the Duke Shared Materials Instrumentation Facility.

**Percoll density gradient analysis of Muc$^T$.** Percoll gradients were prepared by loading 2 mL each of 50%, 30%, and 15% Percoll (prepared in PBS; Cytiva, Marlborough, MA; #17089109) into a 15 mL culture tube, as previously described (8). Bacteria were cultured in the indicated growth medium to stationary phase. Washed bacterial suspensions were added to the Percoll gradients, and tubes were centrifuged (Allegra X-14R Centrifuge, Beckman Coulter, Brea, CA) at 3,000 g for 30 min at 4 °C, with the brake speed set to off or low. Bacteria were collected from the visible layers.

**Analysis of inverton orientation by asymmetrical restriction enzyme digestion.** The CPS inverton upstream of *cps1* in MucT was identified using the long-read inverton predictor PhaVa (9). All publicly available *A. muciniphila* long-read sequencing datasets deposited in the Sequence Read Archive (8 total as of May 2023) were processed through the PhaVa pipeline with *A. muciniphila* ATCC BAA-835 as the reference genome. Primers flanking the putative inverton were used to amplify it (Table S3). Gel-extracted PCR products (QIAquick gel extraction kit, Qiagen, Hilden, Germany) were digested with HpaI (NEB, Ipswich, MA) and run on an agarose gel.

**Analysis of the frequency of inverton orientations by qPCR.** DNA from human fecal samples was extracted using the QIAamp Fast DNA Stool Mini Kit (Qiagen, Hilden, Germany; #51604) (3). Human samples were obtained from individuals naturally colonized with the strains used for matching in vitro analyses (Akk0096, Akk0580, and Akk0490).

For *A. muciniphila* $Muc^T$, five-point standard curves were based on Qubit quantification of PCR products of each respective inverton orientation. To generate the standards, *A. muciniphila* genomic DNA was extracted from a culture and each orientation of the putative inverton was amplified with Q5 High-Fidelity 2X Master Mix (NEB, Ipswich, MA) using the primers in Table S3. The amplicons were extracted from an agarose gel using the QIAquick gel extraction kit (Qiagen, Hilden, Germany), per the manufacturer's recommendations. Quantitative PCRs were run on a QuantStudio 6 Pro real-time PCR system (Applied Biosystems, Waltham, MA) with PowerUp SYBR Green Master Mix

(Thermo Fisher Scientific, Waltham, MA) using the primers in Table S3. Absolute quantification of each orientation was calculated and orientations were plotted as a percentage of total invertons detected.

Putative invertons in other *Akkermansia* strains were identified by manually searching genomes adjacent to *cps* loci for inverted repeat sequences consistent with the motif identified by Jiang and colleagues (CGGATT..AATCCG) (10). For *A. massiliensis* and *A. biwaensis*, reactions were performed with PowerUp SYBR Green Master Mix (Thermo Fisher Scientific, Waltham, MA) using primers listed in Table S3. Relative quantification of orientation was calculated using the comparative Ct method ($RQ=2^{-\Delta CT}$) based on levels of one of the inverton orientations. The percentage of each orientation detected was plotted. “ON” or “OFF” assignment of the respective inverton orientation was based on the predominant inverton orientation observed in vitro and the presence or absence of CPS in vitro.

**Mouse colonization with *A. muciniphila* Muc$^T$ to assess inverton orientations.** Eight-week-old C57BL/6J mice (males N=3, females N=4; Jackson Labs, Bar Harbor, ME; Fig. 4E “Akk gavage”) received antibiotics (1 mg/mL ampicillin, 5 mg/mL streptomycin, 1 mg/mL colistin, 0.25 mg/mL vancomycin, 1% sucrose) in drinking water for 2 weeks to clear endogenous *Akkermansia*, confirmed by PCR (primers in Table S3). Antibiotics were removed 2 days before overnight fasting, and mice were gavaged with 100 µL of *Akkermansia*-free fecal slurry (0.03 g/mL) prepared from *Akkermansia*-free C57BL/6 mice (bred in-house (1)) by homogenization in PBS and centrifugation (10,000g, 3 min). One

day later, mice were fasted overnight and inoculated with *A. muciniphila* Muc$^T$. Cultures grown 36 h in synthetic medium were pelleted (10,000g, 5 min), resuspended in PBS + 25% glycerol, and stored at -80C. Mice received $10^9$ CFU/mouse, using OD600 conversion factor of $4.2\times10^8$ CFU/mL (synthetic) per OD600=1. Fecal pellets were collected 1, 14, and 28 days post-gavage.

**Mouse colonization by vertical transmission with *A. muciniphila* Muc$^T$ for analysis of CPS inverton orientation along the GI tract.** To generate a mouse colony stably colonized with *A. muciniphila* Muc$^T$, in-house-bred *Akkermansia*-free C57BL/6J mice (1) were orally inoculated with ~$10^9$ CFU/mouse of *A. muciniphila* Muc$^T$. These mice were then used as breeders for a colony (since October 2020), which is maintained in sterile cages with autoclaved water (reverse osmosis) and autoclaved food (LabDiet, St. Louis, MO; #5K67). The colony is routinely tested for *Akkermansia* presence using *Akkermansia*-specific primers (Table S3). Intestinal contents from 18-week-old male C57BL/6 mice (N=3; Fig. 4E "*Akk* stably colonized") were obtained by gently scraping off contents with a spatula. The ileum corresponds to the distal third of the small intestine. The colon was divided into proximal and distal halves.

**Mouse colonization with wild-type or CPS$^-$ *A. muciniphila* Muc$^T$.** Nine-week-old *Akkermansia*-free C57BL/6J mice (1) were fasted in the morning and then orally inoculated in the afternoon with ~$10^8$ CFU/mouse of either wild-type or CPS$^-$ (*wzy*::Tn *gad*::Tn) *A. muciniphila* Muc$^T$ or PBS alone (vehicle control) (Fig. S4B-C, 5, 6A-B). Animals of both sexes were used; sex was not considered a biological variable. To

prepare the inocula, *Akkermansia* grown in synthetic+mucin media was centrifuged at 10,000g for 5 minutes. The bacterial pellets were resuspended in sterile PBS to a concentration of $10^9$ CFU/mL, using a conversion factor of $2.7\times10^9$ CFU/mL (mucin) per OD600=1. Fecal and tissue samples were collected 3 weeks post-colonization. Small intestine samples were obtained from the distal third of the small intestine. Colon samples were collected from the distal half of the colon.

**Confocal microscopy.** Tissues were fixed in two washes of Carnoy's solution (60% methanol, 30% chloroform, 10% acetic acid; overnight, RT), washed in 100% ethanol, cleared in xylene (2 × 15 min), paraffin-embedded, and sectioned at 5 µm. Sections were dewaxed (Histo-Clear, Electron Microscopy Sciences, Hatfield, PA; #64110; 2 × 10 min), rehydrated through graded ethanol (100%, 95%, 70%, $dH_2O$), and rinsed in PBS. Slides were blocked with 2% BSA (30 min, RT).

Rabbit anti-*Akkermansia* antisera (generated (1) and purified in-house; Bio-Rad, Hercules, CA; #161-4013(1); 1:100), rat anti-mouse EpCAM (Thermo Fisher Scientific, Waltham, MA; #14-5791-81; 1:500), and UEA-1 Fluorescein (Vector Laboratories, Newark, CA; FL-1061; 1:500) were applied overnight at 4°C in 2% BSA, followed by three PBS washes and secondary antibodies (Thermo Fisher Scientific, Waltham, MA; #A-21428, #A-21247; 1:1000, 30 min, RT). Nuclei were stained with Hoechst (1 µg/mL, 5 min; Thermo Fisher Scientific, Waltham, MA; #H1398), and slides were mounted in Mowiol 4-88 (Sigma-Aldrich, St. Louis, MO; #81381). Images were acquired on a Zeiss LSM 880 equipped with an Airyscan SR for high-resolution imaging (Zeiss, Oberkochen,

Germany). Images were processed using ImageJ. All slides were analyzed under single-blinded conditions (Muc$^{T}$, n = 5; CPS$^{-}$, n = 10). For small intestine analyses, images were selected based on consistent mucus distribution between adjacent villi identified by UEA-1 staining, and fields were chosen to maintain comparable villus length across samples. Up to five villi per image were randomly selected, and perpendicular measurements were taken from the villi base to the nearest detectable *Akkermansia* signal that colocalized with UEA-1. These individual distances were recorded and reported.

For colonic colocalization analyses, images were selected based on the presence of a continuous, intact inner mucus layer labeled with UEA-1. Images were then uniformly cropped to a consistent linear region of interest. Colocalization analysis was performed in ImageJ using the JaCOP plugin, and the resulting colocalization coefficients are reported.

For lumen aggregation analyses, images were selected from luminal fecal pellet contents and required to show a consistent UEA-1 signal. Image processing was performed in ImageJ, where binary open and close operations were applied to reduce noise, followed by thresholding to exclude large background debris. Particle analysis was then conducted using the Analyze Particles function with a minimum size of 0.25 $\mu m^2$ and a circularity range of 0.00 to 1.00.

**Quantification of biofilm formation via crystal violet assay.** Saturated cultures (N=4 per strain; OD600~1.4) of synthetic-grown wild-type or CPS$^{-}$ *A. muciniphila* Muc$^{T}$ were

diluted 1:1 into fresh media and incubated for an additional 3 hours. Two to three wells per inoculum were set up in tissue-culture-treated 96-well plates (Genesee Scientific, Morrisville, NC; #25-109), with 25uL of culture added to 180uL of synthetic media. Plates were sealed with foil and incubated anaerobically at 37C for 48 hours. Wells were mixed (3 pipette strokes), washed with PBS, dried at 37C for 10 min, and stained with 0.1mL of 0.2% crystal violet for 30 min at 37C. After two PBS washes, crystal violet was extracted with 0.2mL of 80% ethanol–20% acetone, and 0.1mL was transferred for absorbance measurement at 600nm in a µQuant plate reader (Bio-Tek, Winooski, VT).

**Propidium iodide staining for biofilm analysis and confocal imaging.** Saturated cultures (OD600 ~1.4) were diluted 1:1 in fresh synthetic media and grown in glass-bottom chamber slides (Thermo Fisher Scientific, Waltham, MA; #177402). After 48 hours, wells were gently mixed and washed once with 200 µL of PBS to remove planktonic cells. Biofilms were stained with 100 µL of propidium iodide (PI) (Thermo Fisher Scientific, Waltham, MA; #R37169) for 15 min at RT in the dark, then washed twice with PBS. Images were acquired on a Zeiss LSM 880 confocal microscope equipped with Airyscan SR for high-resolution imaging (Zeiss, Oberkochen, Germany).

## REFERENCES FOR SUPPLEMENTAL MATERIALS AND METHODS

1. Davey LE, Malkus PN, Villa M, Dolat L, Holmes ZC, Letourneau J, Ansaldo E, David LA, Barton GM, Valdivia RH. 2023. A genetic system for Akkermansia muciniphila reveals a role for mucin foraging in gut colonization and host sterol biosynthesis gene expression. Nat Microbiol 8:1450-1467.
2. Derrien M, Vaughan EE, Plugge CM, Vos WMd. 2004. Akkermansia muciniphila gen. nov., sp. nov., a human intestinal mucin-degrading bacterium. International Journal of Systematic and Evolutionary Microbiology 54.

3. Becken B, Davey L, Middleton DR, Mueller KD, Sharma A, Holmes ZC, Dallow E, Remick B, Barton GM, David LA, McCann JR, Armstrong SC, Malkus P, Valdivia RH. 2021. Genotypic and Phenotypic Diversity among Human Isolates of Akkermansia muciniphila. mBio 12.
4. McCann JR, Bihlmeyer NA, Roche K, Catherine C, Jawahar J, Kwee LC, Younge NE, Silverman J, Ilkayeva O, Sarria C, Zizzi A, Wootton J, Poppe L, Anderson P, Arlotto M, Wei Z, Granek JA, Valdivia RH, David LA, Dressman HK, Newgard CB, Shah SH, Seed PC, Rawls JF, Armstrong SC. 2021. The Pediatric Obesity Microbiome and Metabolism Study (POMMS): Methods, Baseline Data, and Early Insights. Obesity (Silver Spring) 29:569-578.
5. Eddy SR. 2011. Accelerated Profile HMM Searches. PLoS Comput Biol 7:e1002195.
6. Sievers F, Wilm A, Dineen D, Gibson TJ, Karplus K, Li W, Lopez R, McWilliam H, Remmert M, Soding J, Thompson JD, Higgins DG. 2011. Fast, scalable generation of high-quality protein multiple sequence alignments using Clustal Omega. Mol Syst Biol 7:539.
7. Letunic I, Bork P. 2021. Interactive Tree Of Life (iTOL) v5: an online tool for phylogenetic tree display and annotation. Nucleic Acids Res 49:W293-W296.
8. Dorman MJ, Feltwell T, Goulding DA, Parkhill J, Short FL. 2018. The Capsule Regulatory Network of Klebsiella pneumoniae Defined by density-TraDISort. mBio 9.
9. Chanin RB, West PT, Wirbel J, Gill MO, Green GZM, Park RM, Enright N, Miklos AM, Hickey AS, Brooks EF, Lum KK, Cristea IM, Bhatt AS. 2024. Intragenic DNA inversions expand bacterial coding capacity. Nature 634:234-242.
10. Jiang X, Hall AB, Arthur TD, Plichta DR, Covington CT, Poyet M, Crothers J, Moses PL, Tolonen AC, Vlamakis H, Alm EJ, Xavier RJ. 2019. Invertible promoters mediate bacterial phase variation, antibiotic resistance, and host adaptation in the gut. Science 363:181-187.

Figure S1. Synteny analysis of *cps1-3* loci.

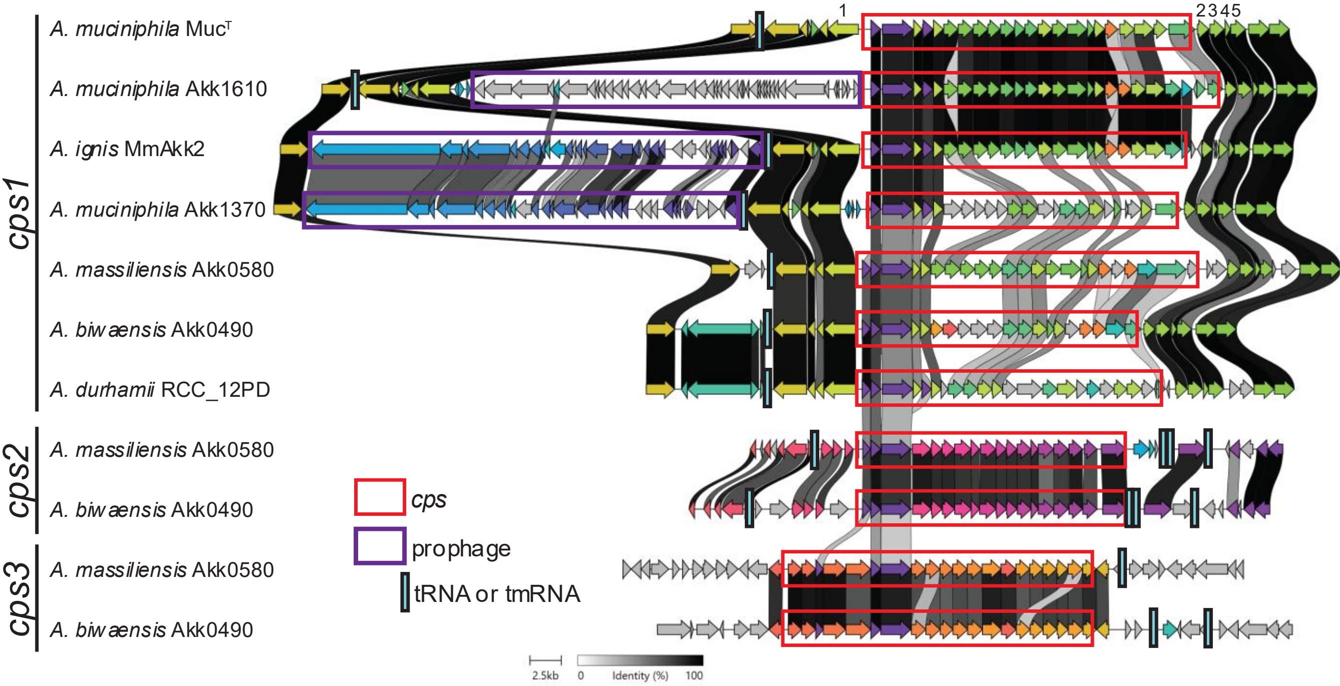

Figure S2. Capsule formation in *A. muciniphila* is Wzy- and Wza-dependent.

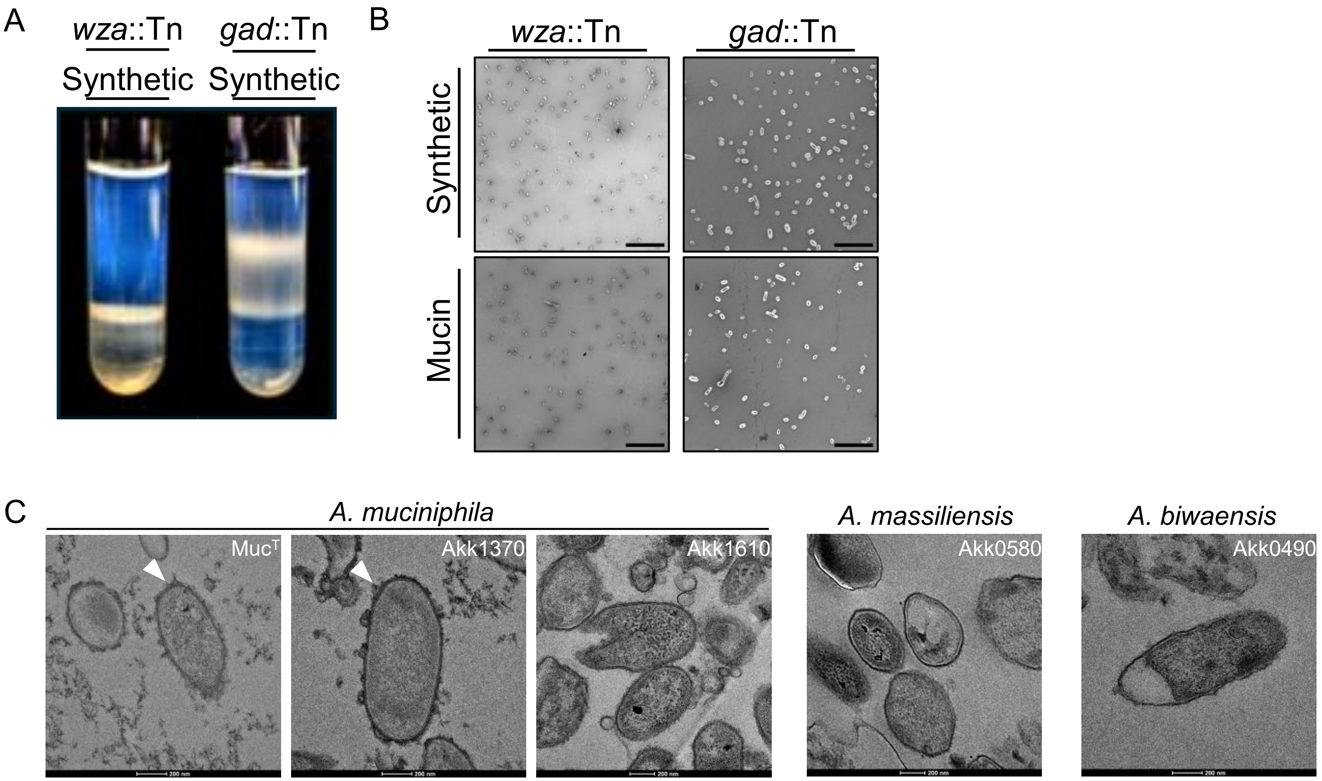

Figure S3. Growth curve for samples used for gene expression analysis.

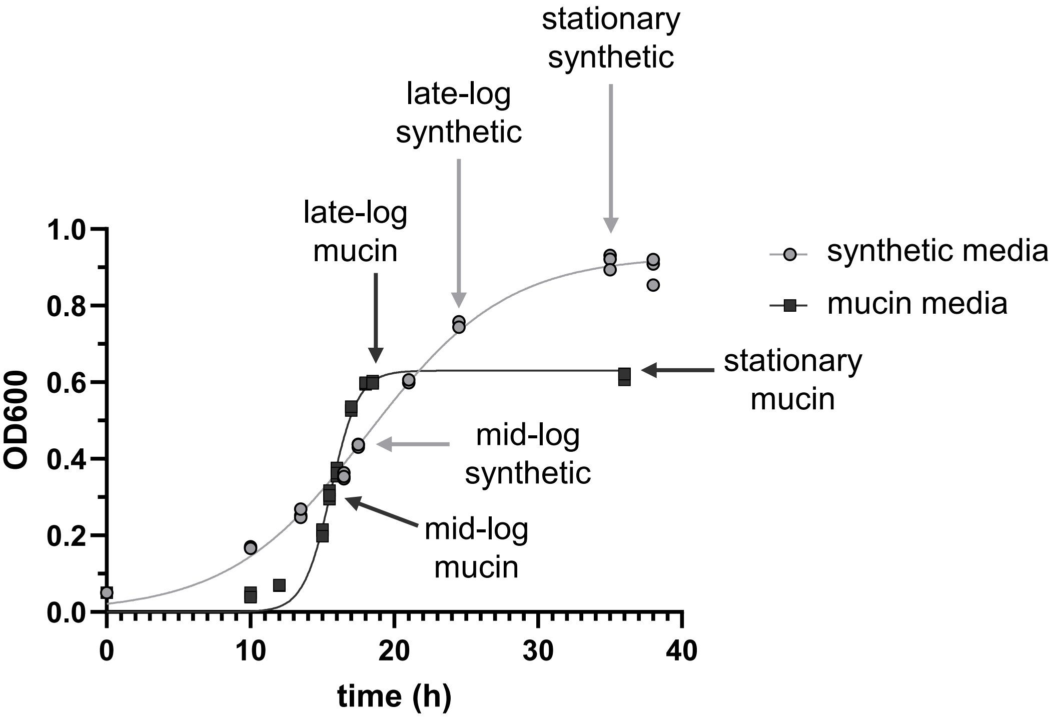

Figure S4. INSeq analysis and single colonization experiments of mice with *A. muciniphila*.

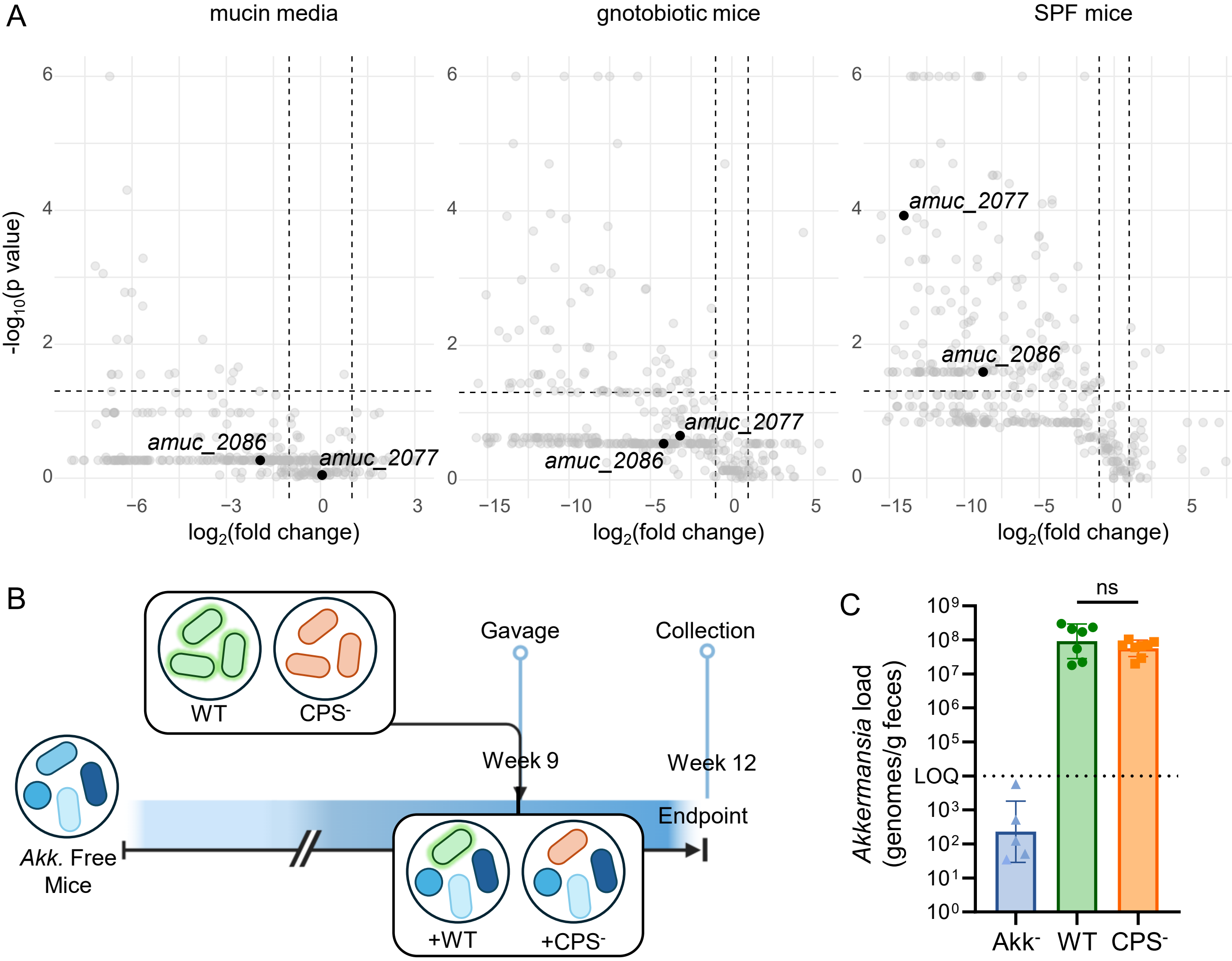

Figure S5. Growth kinetics of *A. muciniphila* in the presence of LL-37.

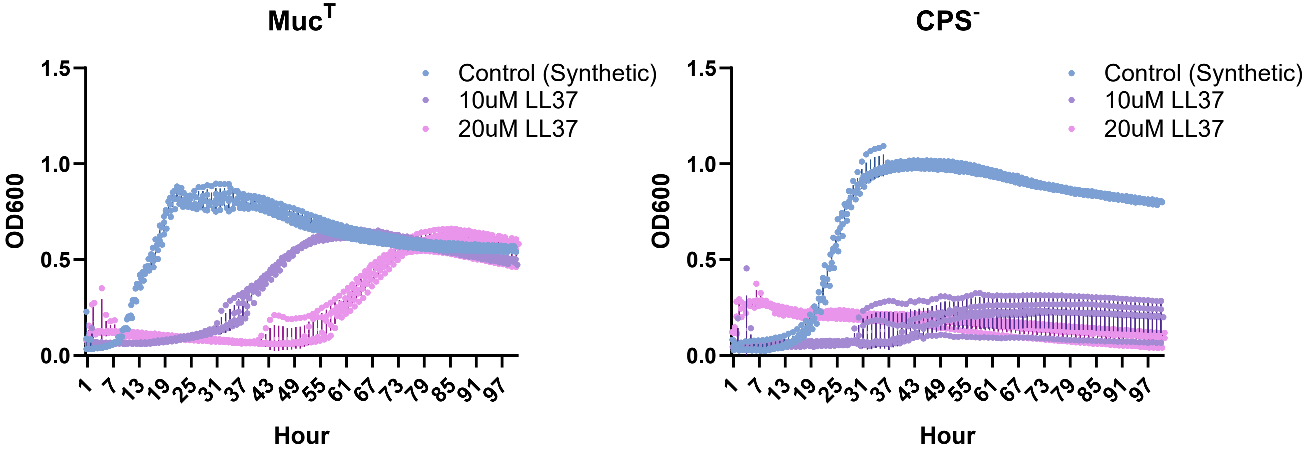

Supplement: Supplemental Material — Supplemental figures and expanded methods. [file mbio.01048-26-s0001.pdf]
